# Scanning tip measurement for identification of point defects

**DOI:** 10.1186/1556-276X-6-140

**Published:** 2011-02-14

**Authors:** László Dózsa, György Molnár, Vito Raineri, Filippo Giannazzo, János Ferencz, Štefan Lányi

**Affiliations:** 1 Research Institute for Technical Physics and Materials Sciences, P.O. 49, H-1525 Budapest, Hungary; 2 CNR-IMM, Strada VIII 5, 95121 Catania, Italy; 3 Institue of Physics, Slovakian Academy of Sciences, Dúbravská cesta 9, SK-845 11 Bratislava, Slovakia

## Abstract

Self-assembled iron-silicide nanostructures were prepared by reactive deposition epitaxy of Fe onto silicon. Capacitance-voltage, current-voltage, and deep level transient spectroscopy (DLTS) were used to measure the electrical properties of Au/silicon Schottky junctions. Spreading resistance and scanning probe capacitance microscopy (SCM) were applied to measure local electrical properties. Using a preamplifier the sensitivity of DLTS was increased satisfactorily to measure transients of the scanning tip semiconductor junction. In the Fe-deposited area, Fe-related defects dominate the surface layer in about 0.5 μm depth. These defects deteriorated the Schottky junction characteristic. Outside the Fe-deposited area, Fe-related defect concentration was identified in a thin layer near the surface. The defect transients in this area were measured both in macroscopic Schottky junctions and by scanning tip DLTS and were detected by bias modulation frequency dependence in SCM.

## Introduction

Nanostructures require investigation of local electrical characteristics with high spatial resolution [1]. Non-destructive measurement of the surface and the interfaces is critical in SOI materials [2], such techniques are technologically important in characterization of growth processes [3] and in measurement of dielectric layers [4]. Defect identification was investigated in detail using few millimeter size electrodes [5]. Metal silicide films have attracted attention because of their scientific curiosity and technical importance [6]. Fe is a critical contamination in silicon and investigation of the defects related to Fe is technologically important. In earlier studies we have investigated microscopic, structural, and electric properties of FeSi_2 _layers [7-10]. Noise and deep level transient spectroscopy (DLTS) investigation of β-FeSi_2 _quantum dots embedded in silicon show that Schottky junctions are not effective in evaluating defects in the Fe-Si system since the device current is described by space charge limited current and the depleted layer model is not applicable [11]. Scanning probe capacitance microscopy (SCM) was applied to measure the local electrical characteristics; however, the isolated quantum dots could not be resolved due to the large concentration of Fe-related defects. The results show that for understanding the electrical properties of nanostructures the measurement of electric transport on nanoscale is necessary. In an earlier study we have shown that the SCM transient on the silicon surface near the Fe-contaminated region indicates surface contamination [7].

In this study we identify defects outside the Fe-deposited region by DLTS and demonstrate the possibility of nanoscale defect identification by scanning tip DLTS. It is shown that SCM modulation frequency dependence properly indicated point defects.

### Sample preparation and measurements

*N*-type (100)-oriented Si wafers were used as substrates. The backside was implanted by P^31^+ (40 keV, 480 μC), cleaned by plasma and wet cleaning processes and annealed at 900°C for 30 min in N_2 _ambient. Before loading the samples into the UHV evaporation chamber, their surface was refreshed in diluted HF. The time elapsed after cleaning to reach 1 Pa pressure in the UHV chamber was about 30 min. After evacuation down to 1 × 10-6 Pa and prior to evaporation, Si wafers were annealed in situ for 5 min at 800°C. Iron has been evaporated from ingots of 99.9% purity using an electron gun at a pressure of 3 × 10-6 Pa by RDE process at 0.015 nm/s rate onto the 600°C substrate, and further annealed for 5 min at the same temperature. Contamination was indicated outside the Fe deposition by SCM [7]. To identify this contamination by DLTS half of the Si wafer fragment was covered during Fe deposition and 400 μm × 400 μm rectangular Au dots Schottky junctions were prepared both in and outside the Fe-deposited area.

SCM was measured by a DI 300 nanoscope equipped with scanning capacitance facility. The capacitance is measured at 1 GHz. The local *dC*/*dV *is measured using lock-in technique with bias modulation in the 5-120 kHz range [7]. The heavily doped silicon tip covered by a thin diamond layer, an air gap, and the conductive substrate are evaluated as a simple MOS structure. In this approximation the measured local *dC*/*dV *is usually interpreted as dopant concentration under the tip. *C*-*V*, *I*-*V*, and DLTS characteristics were measured in a SEMILAB 83D system. A preamplifier was developed for the capacitance input of the DLS83D equipment. A 100-nm radius tungsten tip connected directly to the preamplifier input was positioned above the silicon surface. The apparent capacitance was amplified 200 times as it was calibrated by measuring 1 and 5 pF standard capacitances with and without the preamplifier. The amplification of capacitance without increased noise is possible since the low noise preamplifier decouples the load of the measuring cables from the measuring tip. This method will be referred to as scanning tip DLTS.

The spreading resistance (SR) was measured in an SSM130 system using two measuring tips at 100 μm distance. A series of clean silicon with doping in the 0.08-180 Ω cm range were used to calibrate the SR measurement.

## Results

### Electrical characteristics of Schottky junctions

The *I*-*V *characteristics of Schottky junctions in the Fe-deposited area at room temperature were dominated by the series resistance and leakage. The series resistance on the Fe-deposited area has a high scatter. The Schottky junction prepared on silicon outside the Fe-deposited area has 0.73 V built-in voltage and is appropriate for identification of defects by DLTS. SR measurements were carried out on beveled samples of the Fe-deposited area. It shows that the resistivity in the Fe-deposited region is an order of magnitude higher in about 0.3-0.4 μm depth. The resistivity in the Fe-deposited region has shown large scatter. The silicon in about 1 μm depth below the Fe deposition exhibits resistivity appropriate for the silicon starting wafer.

**Figure 1:**
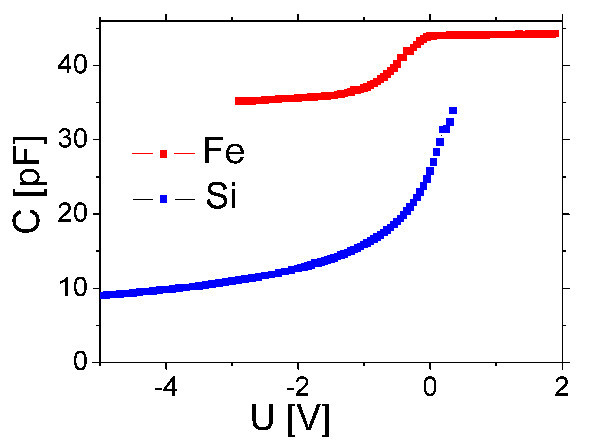
***C*-*V *characteristics of Schottky junction prepared in the Fe-deposited area (Fe) and on the silicon surface (Si)**.

The *C*-*V *characteristics of a Schottky junction prepared on the Fe-deposited area and on the free silicon are shown in Figure 1. The capacitance of the Schottky junction on the Fe-deposited area is larger, indicating that the Fe-generated defect concentration in this area in about 0.5 μm depth is few time 10^16^/cm^3^. This defect concentration is an order of magnitude larger than the 2 × 10^15^/cm^3 ^doping determined from the 1/*C*^2^-*V *plot measured in junctions prepared on the silicon surface. These defects are donor type deep level defects, situated about 250-300 meV below the conduction band edge.

### Scanning capacitance measurements

**Figure 2:**
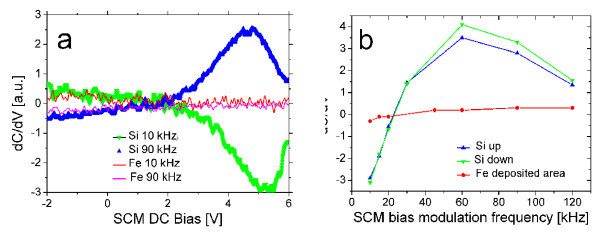
**SCM signal measured on the Fe-deposited area and on the silicon surface**. **a. ***dC*/*dV*-voltage plots at 10 and 90 kHz bias. **b. **Dependence of the amplitude of the *dC*/*dV *peak at about +5 V on the bias modulation frequencies.

In the Fe-deposited area SCM has shown a patterned surface on the 1 μm scale. The SCM contrast was clear; however, comparison of SCM images with secondary electron microscopy and AFM images shows that the quantum dots cannot be resolved by SCM [7]. A summary of the SCM measurements is shown in Figure 2. *dC*/*dV*-bias characteristics at 10 and 90 kHz modulation frequencies are shown in Figure 2a. The bias dependence of *dC*/*dV *in the Fe-deposited area is weak as is shown in Figure 2a. Outside the Fe-deposited area the sign of the peak in *dC*/*dV *has changed by varying the bias modulation frequency from 10 to 90 kHz as it is shown in Figure 2a. The position of the peak at about 5 V bias was independent of the modulation frequency, however, its amplitude varied with bias modulation frequency as it is shown in Figure 2b. The scans repeated with increasing and decreasing bias modulation frequencies exhibited some hysteresis indicated by Si up and Si down in Figure 2b. The change in the sign of *dC*/*dV *can be explained by defects on the surface.

### DLTS measurements

DLTS identified the Fe-related defects in the Fe-deposited area; however, weak junction characteristics prevent proper evaluation of the defects by DLTS. *C*-*V *profile has shown that in the Fe-deposited area the deep defect concentration is few times 10^16^/cm^3^, order of magnitude larger than the doping of the starting silicon wafer in about 0.5 μm depth, which does not suit for DLTS measurements.

**Figure 3:**
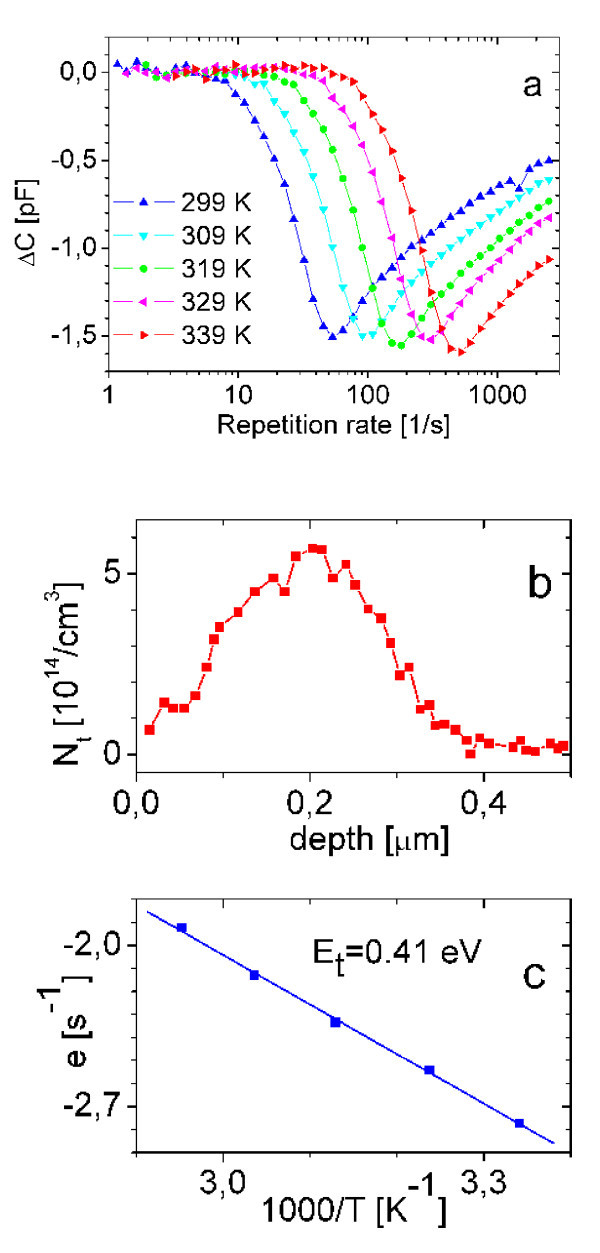
**DLTS results in macroscopic Schottky junctions**. **a. **DLTS frequency scan spectra measured by DLS-83D system. **b. **Depth profile of the defect. **c. **Arrhenius plot for determination of the activation energy of the defect.

DLTS spectra measured in a Schottky junction outside the Fe deposition area are shown in Figure 3a. The spectra were recorded at -5 V reverse bias and 0 V bias, 5 μs filling pulses. The spectra are broad, indicating that the defect activation energy is distributed. The depth profile measured by DLTS is shown in Figure 3b. The defects are localized at about 200 nm from the silicon surface. The activation energy of the defect determined by Arrhenius plot shown in Figure 3c agrees with a defect attributed to Fe in silicon [12]. We remark that the depth profile of the defect may be also interpreted as a distributed energy surface state in 5 × 10^10^/cm^2 ^density, since the depth resolution of the capacitance DLTS technique is not satisfactory to distinguish these details.

### Scanning tip DLTS measurements

A measuring tip was shaped from an 80-μm diameter tungsten wire to an approximately 100 nm radius. It was placed in a shield extending to about 0.5 mm from the surface, to reduce the stray capacitance. The tip was positioned as near as possible without measurable current (few pA) through the tip-wafer junction. It represents an MIS structure. In the Fe-deposited area the tip-wafer capacitance did not depend on the applied bias and no DLTS signal was detected. It is explained by the high defect concentration surface layer.

**Figure 4:**
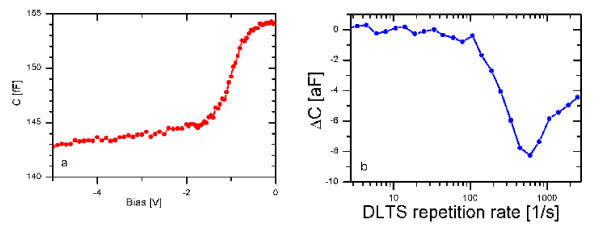
**Electrical characteristics measured by the capacitance preamplifier with a 100 nm radius tip positioned near the silicon surface**. **a. ***C*-*V *characteristics. **b**. DLTS frequency scan.

The measured scanning tip *C*-*V *characteristic outside the Fe deposition is shown in Figure 4a. The scanning tip DLTS frequency scan spectrum measured in the same position is shown in Figure 4b. The spectra were measured at room temperature. The spectra measured in different position on the surface show a scatter in amplitude and peak position. This property is analogous to the large scatter observed by SCM outside the Fe deposition area, and the scatter of the DLTS spectra measured in different position Schottky junctions.

## Discussion

The above results were measured in a wide frequency range from DC to 1 GHz range, and with contact size from few tens of nanometers to 400 μm. SCM capacitance measured at 1 GHz is limited by the relaxation time of defects. Only the free carriers in the bulk silicon can follow this excitation. For this reason the deep-level defects can be detected only on the free silicon surface, since defects in the Fe-deposited region have much larger time constant than 1 ns. The Fe-related defects may influence the space charge in the 5-120 kHz modulation frequency range at room temperature. SCM detects an electrically overlapping network of conductive quantum dots on the Fe-deposited area. The capacitance measurements at 1 MHz indicate an about 0.5 μm wide defective layer on the Fe-deposited area. The defective layer is due to large concentration of Fe-related defects which can follow only the low frequency excitation. In the capacitance transient measurements (1 MHz in DLTS and 1 GHz in SCM) these defects do not follow the bias modulation, but these defects dominate the steady state *I*-*V*, *C*-*V*, and SR measurements.

On the free silicon surface at low bias modulation frequency (below 20-30 kHz) in SCM *dC*/*dV *the Fe-related defect can follow the modulation but at higher frequency only the free carriers follow the excitation, and *dC*/*dV *gives *n*-type silicon doping. It is analogous to the admittance spectroscopy in macroscopic junctions [13,14]. The scanning tip DLTS spectra are similar to large area Schottky junctions. The results demonstrate that SCM and scanning tip DLTS are able to identify defects in semiconductors with high spatial resolution.

## Conclusion

FeSi_2 _quantum dots were grown by in situ self-organized growth process on silicon. In the Fe-deposited layer a resistive layer with high concentration of defects dominates the characteristics; charge captured on these defects can follow only the low frequency modulation. The concentration of the Fe-related deep-level defects generated outside the Fe-deposited region was found in about 5 × 10^14^/cm^3 ^concentration near the surface. These Fe-related defects are localized at about 200 nm depth from the surface and may be interpreted also as distributed energy surface state defects in about 2 × 10^10^/cm^2 ^concentration. Scanning tip capacitance DLTS spectra on the free silicon surface are analogous to those measured in Schottky junctions. The defects are indicated by the modulation frequency dependence of the SCM *dC*/*dV *signal, showing a tool to detect point defects on microscopic scale by SCM.

## Abbreviations

DLTS: deep level transient spectroscopy; SCM: scanning probe capacitance microscopy; SR: spreading resistance.

## Competing interests

The authors declare that they have no competing interests.

## Authors' contributions

FG and VR carried our the SCM experiment, GM prepared the investigated structures and participated in the plan of the study, SL participated in the design and has built the preamplifier and piezo positioner, JF measured the spreading resistance, LD measured DLTS and participated in the design of the preamplifier and plan of study
